# Visual chunking as a strategy for spatial thinking in STEM

**DOI:** 10.1186/s41235-020-00217-6

**Published:** 2020-04-18

**Authors:** Mike Stieff, Stephanie Werner, Dane DeSutter, Steve Franconeri, Mary Hegarty

**Affiliations:** 1 University of Illinois-Chicago, Chicago, IL USA; 2 Northwestern University, Evanston, IL USA; 3 University of California-Santa Barbara, Santa Barbara, CA USA

**Keywords:** Visual Memory, Expertise, Spatial skills

## Abstract

Working memory capacity is known to predict the performance of novices and experts on a variety of tasks found in STEM (Science, Technology, Engineering, and Mathematics). A common feature of STEM tasks is that they require the problem solver to encode and transform complex spatial information depicted in disciplinary representations that seemingly exceed the known capacity limits of visuospatial working memory. Understanding these limits and how visuospatial information is encoded and transformed differently by STEM learners presents new avenues for addressing the challenges students face while navigating STEM classes and degree programs. Here, we describe two studies that explore student accuracy at detecting color changes in visual stimuli from the discipline of chemistry. We demonstrate that both naive and novice chemistry students’ encoding of visuospatial information is affected by how information is visually structured in “chunks” prevalent across chemistry representations. In both studies we show that students are more accurate at detecting color changes within chemistry-relevant chunks compared to changes that occur outside of them, but performance was not affected by the dimensionality of the structure (2D vs 3D) or the presence of redundancies in the visual representation. These studies support the hypothesis that strategies for chunking the spatial structure of information may be critical tools for transcending otherwise severely limited visuospatial capacity in the absence of expertise.

## Significance statement

Spatial thinking is critical for learning and problem-solving in STEM disciplines. Extant studies show that spatial skills are important for visuospatial thinking in the sciences, but the cognitive processes that underlie spatial thinking remain poorly understood. This paper advances our understanding of how novice chemistry students, as well as students naive to chemistry, perceive, encode, and transform spatial information in the domain. Our work identifies how novice and naive STEM learners can exceed the limits of visual working memory capacity to encode spatial information in disciplinary representations. This research provides insight into the early stages of expertise development in a science domain, such as chemistry, and it has the potential to reveal which types of future educational interventions are most likely to be effective.

## Background

Spatial thinking is a core component of scientific practice (National Research Council, 2006). In all science disciplines, individuals must identify spatial relationships and make predictions about them. To do so, they must reason about spatial concepts that include size, distance, and position and how these concepts change over time. Spatial thinking often tasks scientists with making precise estimates of the expected location of an object after one or more spatial transformations. These estimates range from a few transformations of simple shapes, such as the lateral movement of a ball across a plane, to many transformations of complex shapes, such as the folding of a protein.

Across such examples, experts display a remarkable ability to encode and retrieve large amounts of spatial information and to simulate transformations of that information with high fidelity. Yet, the mechanisms by which experts accomplish these difficult tasks remain poorly understood. Even less understood are the processes that STEM novices and students naive to STEM rely on as they begin their journey toward expertise. Arguably, our understanding is lacking given that much of the research on spatial thinking in science domains has focused on the role of individual differences in spatial skills as predictors of achievement in STEM disciplines without attending to the cognitive processes engaged when encoding and retrieving spatial information (Uttal et al., 2013; Wai, Lubinski, & Benbow, 2009). While this effort has demonstrated that spatial skills are sometimes correlated with performance in STEM, it has provided little about how or why experts excel at visuospatial thinking or how novices begin to develop expert strategies for spatial thinking.

Outside of science domains, research on the nature of expertise and its development has shown that the superior performance of experts results not from their larger mental capacity or their leveraging of domain general processes typically associated with individual differences in constructs (such as spatial visualization), but in their accumulation of domain knowledge (Alexander, 2013) and their increased sensitivity to perceive and encode recurring patterns of information in the environment (Kellman & Massey, 2013). This ability to quickly detect and encode patterns allows experts to exceed typical estimates of visual working memory capacity and achieve superior performance by leveraging a form of information compression. This feat, however, is highly constrained by domain knowledge. For example, expert chess players can encode and retrieve vast numbers of chessboard configurations but only if these configurations are legal according to gameplay rules. When presented with illegal configurations, experts perform no differently from novices with respect to memory for the number or location of chess pieces (Chase & Simon, 1973; Robbins et al., 1996). The specificity of expertise for the encoding and retrieval of domain-relevant information has also been observed among various fields, such as mathematics and social science (Baroody, 2003; Voss, Tyler, & Yengo, 1983). In sum, the increased visual working memory capacity of experts does not seem to be driven by an ability to encode more information than novices. Instead, the experts seem to leverage their knowledge and their history of repeated exposure to domain-relevant patterns, allowing them to process and store complex *chunks* of information.

While spatial skills are correlated with greater spatial working memory capacity (Shah & Miyake, 1996) and with science achievement (Wai et al., 2009), whether successful science novices have superior ability to transform spatial information (as measured by tests of spatial skills) or a proclivity to notice recurring patterns of spatial information in science representations similar to that of experts in nonscience domains, such as chess, remains unclear. Here, we explore the relationship between spatial skills and visual working memory capacity in the context of chemistry. We show that novice chemistry students, as well as students naive to chemistry, are sensitive to the structure of information embedded in disciplinary representations despite lacking significant domain knowledge. We argue that naive and novice students can readily notice recurring patterns in disciplinary representations: patterns that will eventually be identified as meaningful “conceptual chunks” by experts. This simple repeated exposure to recurring patterns in scientific representations should allow students with both high and low spatial skills to exceed the typical limits of visual working memory capacity by isolating and learning the structure of a chunk even though they lack the significant semantic knowledge of an expert. This finding contributes to our understanding of how expertise in visuospatial thinking develops in science domains and offers implications for improving STEM education more broadly.

### Visual working memory capacity and the expertise effect

Working memory capacity is defined as the number of memory representations that an individual can activate and focus on at a given time (Engle, 2002). The limits of working memory and the conditions under which these limits can be exceeded have been comprehensively studied. The upper limit of visual working memory capacity is generally accepted as being, at most, four representations (Alvarez & Cavanagh, 2004; Unsworth & Engle, 2007); however, whether this capacity is best defined as a limit on independent representations (Zhang & Luck, 2008) or as more-complex linked structures has been debated (Brady, Konkle, & Alvarez, 2011). The reported capacity estimates seem low and would suggest that both experts and novices should struggle with maintaining high fidelity representations of complex displays in memory. But the granularity of a memory representation can vary greatly with the resolution of a given representation and varies with expertise (Scolari, Vogel, & Awh, 2008). This relationship ostensibly contributes to the seemingly large working memory capacity of experts: While experts, like novices, can maintain roughly the same number of representations in working memory, expert representations are significantly more complex when they involve domain knowledge. Consequently, experts can focus on a larger amount of information than novices, allowing their superior response time, recall, and problem-solving.

**Fig. 1:**
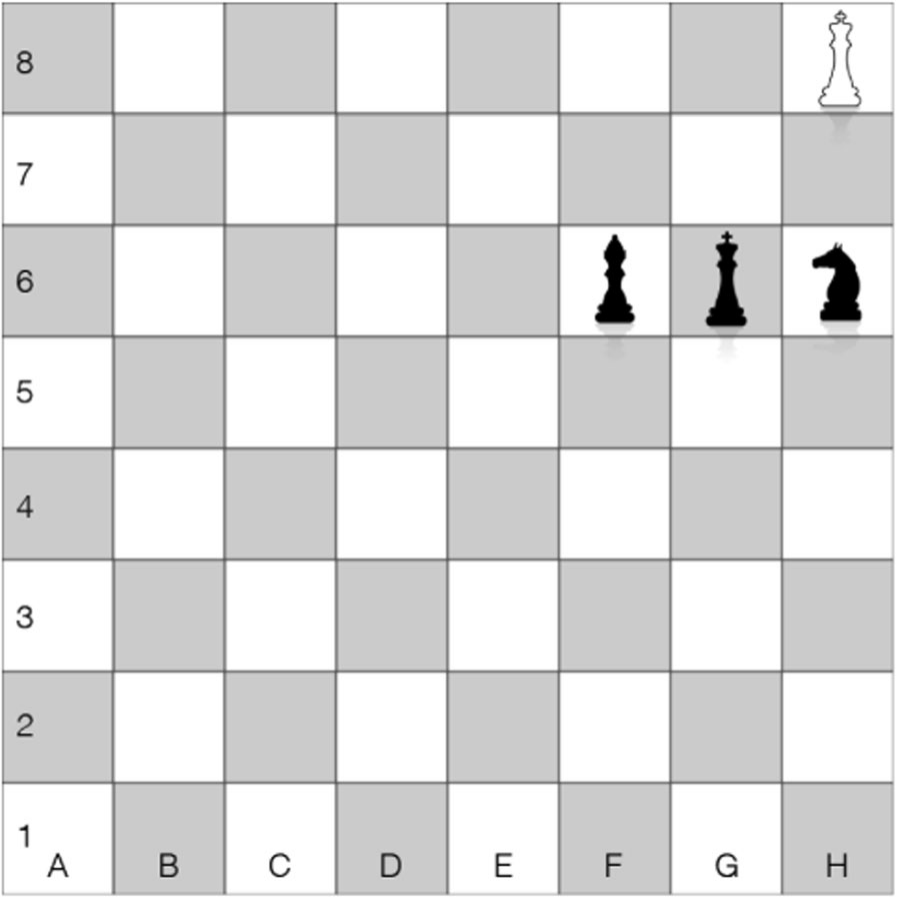
Chessboard configuration of bishop and knight mate checkmate pattern

Although the role of spatial skills in expert performance has been studied extensively in science domains (Uttal et al., 2013), the role of visual working memory capacity in the development of expertise has received less attention (Curby, Glazek, & Gauthier, 2014). Research on the nature of expertise in a knowledge domain has demonstrated that experts can encode and recall large amounts of information in a complex visual display as long as the display is relevant to their expertise (Chase & Simon, 1973). When viewing domain-relevant stimuli, experts do not encode individual components of the stimuli as separate memory representations. Rather, they encode the components and the relationships between the components as a single representation (Scolari et al., 2008) through a process referred to as “chunking” information (Miller, 1956). This feat is illustrated with an example from chess. The layout of the chess pieces in Fig. 1 correspond to a “bishop and knight mate” checkmate pattern. While a chess novice might encode the image as four separate representations that contain identity and location features (*Bf6*, *Kg6*, *Nh6*, and *Kh8*), the expert encodes a single informational “chunk” (*bishop-and-knight-mate*) that contains the identity and relative location features and cues additional semantic knowledge, such as how to avoid or achieve the pattern and the consequence on the game outcome. The maintenance of the single chunk as opposed to the four piece-location pairs reduces the load on working memory and frees additional resources for the expert, which in turn improves task performance (Chase & Simon, 1973).

Research on visual working memory capacity has only more recently begun to focus on performance enhancements due to chunking, with sparse attention to the role of disciplinary expertise. These studies most often measure memory capacity for the *colors* or other visual features of a set of simple objects that are otherwise meaningless to a viewer. Such work typically reports capacity limits of approximately three to four objects (Luck & Vogel, 1997), but this limit drops to approximately one object with additional restrictions, such as remembering the proper position of a single color after a mental rotation operation (Xu & Franconeri, 2015). Most of the visual working memory literature has focused on the capacity of visual working memory—how *many* colors or other visual features a participant can remember for the purpose of detecting changes in a second display—and how this capacity varies with object complexity (for review, see Brady et al., 2011; Suchow, Fougnie, Brady, & Alvarez, 2014). This literature has a new focus on moving beyond capacity for individual colors toward more complex representations that compress visual information.

We define *visual chunking strategies* as any encoding strategy that leverages any aspect of the structure of a set of visual objects that can support the compression of information. Table 1 illustrates three types of visual chunking strategies that a viewer might employ to compress information in a visual display. *Domain-general redundancy* involves compression via the encoding of repeated identities (letters here, colors in the present experiments). *Spatial grouping* involves compression via the encoding of individual units as a single unit based on their spatial proximity to each other. Finally, *expert chunking* involves compression via encoding of patterns of units that are holistically associated in long-term memory by domain experts (e.g., pairs of letters that form words, chess piece configurations, groups of atoms that occur frequently in chemistry structures and have significance (meaning) to chemists). These patterns are inherent to chunks as defined in the expertise literature. Critically, structural regularities in disciplinary representations should allow viewers to compress visual information through any of these strategies. In turn, these strategies should allow viewers to avoid encoding unique identities and instead encode groups, redundancies, and patterns that functionally increase visual working memory capacity. What remains unclear is whether and how novices employ these different strategies as they begin to learn expert strategies.

**Table 1:** Ways to chunk visually presented information. Letter identities can represent different colors, shapes, atomic identities, or any other property

String 1	M Z K	3 units	Not compressible
String 2	M K K	3 units	Compressible to 2 units via *domain-general redundancy*
String 3	M ZK	3 units	Potentially compressible to 2 units via *spatial grouping*, although this grouping does not necessarily help memory performance if no memory association exists between the letters
String 4	M OK	3 units	Compressible to 2 units via spatial grouping and semantic link between O and K available to experts through *expert chunking*
String 5	M KK	3 units	Compressible to 2 units via *domain-general redundancy* and *spatial grouping*

The different strategies can be illustrated using strings of letters, as in the table. An uncompressed set of objects is represented by independently encoded letters in String 1 (“M Z K”). But in all of the other lines of the figure, strategies exist that can be employed by an observer to chunk the three letters using associations between them (Brady & Tenenbaum, 2013). For example, Strings 2 and 3 (“M K K” & “M KK”) each contain one repetition of the letter K that is easy to recognize—any viewer with an understanding of letter identities is likely to see that repetition in this *domain-general redundancy*. That repetition seems even easier to spot when the letters are spatially grouped in the latter example. Chunking this type of redundancy appears to help boost memory capacity in displays of colors, by allowing observers to note the positions of repeated information in a display or implicitly note the mean and variance of the hue histogram (when colors are present) of the collective set of identities (Brady & Alvarez, 2011, 2015a, b). String 4 (“M ZK”) visually groups two of the letters, but this group does not necessarily help memory performance if no memory association exists between the letters Z and K as expected among novices. But this string might encourage a viewer to jointly encode those two items during the development of expertise, which could lead to the types of associations between Z and K that eventually lead it to become a domain-specific pattern. In contrast, String 5 (“M OK”) can be encoded using existing knowledge of a domain-specific pattern: the particular letter combination “OK,” which allows the viewers with knowledge of the English language to see these two letters as not only visually grouped but semantically related.

Various training paradigms have been shown to improve novice performance on visual working memory that involve detecting changes in a stimulus. Recent work shows that such identity chunking allows information compression and higher visual memory capacity in displays of colors where viewers are “taught,” via repeating patterns of information across an experiment, that pairs of colors (e.g., red with blue) tend to co-occur, either within objects or between nearby pairs of objects (Brady, Konkle, & Alvarez, 2009). Memory for complex shapes also improves over the course of a visual memory experiment, as viewers learn to understand the complex features that can and cannot be used to distinguish among objects (Moore, Cohen, & Ranganath, 2006). For example, memory for displays with pairs of colors that co-occur is better than memory for displays with random colors. Thus, perceptual sensitivity for visually grouped information can be improved from both direct instruction and passive viewing.

Despite the extensive research on these strategies for expanding visual working memory capacity, these findings have not been sufficiently linked to real world domains. In contrast, research with domain experts has typically focused on how their domain knowledge helps them perceive more complex patterns but more rarely explores how general attentional strategies are used to bootstrap sensitivity to other types of chunk structure. Ostensibly, the processes and heuristics employed by the cognitive system to encode visual information should form the foundations of, or at least inform, the development of expert chunking strategies. The natural tendency of individuals to perceive regularities (chunk structure) in visual displays suggests that science learners are likely to be sensitive to any of these same types of structure in disciplinary representations. Possibly, being sensitive to structure in disciplinary representations, deliberately or not, may allow the novice learner to bootstrap visual chunking strategies that develop further from extended practice or direct instruction. Alternatively, experts may self-select into a science domain due to an increased general sensitivity for these types of visual structure, which may be related to spatial skills.

### Spatial grouping in scientific representations

In the studies below, we test whether novice chemistry students and students naive to the domain are sensitive to the chunk structure of visuospatial information as they encode and detect changes to domain representations. Chemistry is an ideal discipline to study the development of spatial expertise because a core characteristic of the domain is a reliance on complex visual representations of spatial information for problem-solving. The chemistry curriculum tasks students with learning about the relationships between imperceptible phenomena and using those interactions among these phenomena to reason about the observable properties of matter. These phenomena are inherently visual in nature, which has given chemistry the moniker “the most visual of the sciences” (Habraken, 1996).

Understanding the spatial relationships within and between chemical structures is particularly important because a compound’s structure determines its chemical and physical properties. Even when two molecules contain the same atomic identities (“constituencies” in chemistry) that result in the same chemical formula, the unique connectivity of atoms can produce significant differences in chemical reactivity. So too, small changes in a molecule’s internal three-dimensional spatial arrangement of atoms can result in compounds called stereoisomers that contain the same constituencies yet have unique spatial relationships among atoms that give rise to unique chemical properties. For example, thalidomide, a drug used to treat nausea during pregnancy, was administered to the public as a mixture of two stereoisomers. While one isomer relieved nausea, the other had no therapeutic effect but instead caused serious mutations in the developing fetus. Examples such as these not only show how spatial thinking is central to the discipline but also show that misunderstanding how spatial relationships impact chemical and physical properties can have drastic consequences in applied settings.

**Fig. 2:**
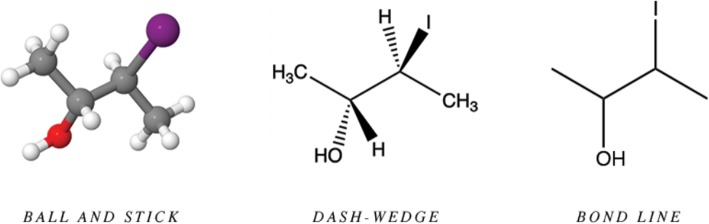
Equivalent representations of (2R,3S)-3-iodobutan-2-ol

Like other scientists, chemists use a variety of visual representations to communicate and solve problems. These representations vary substantially in how spatial information is made explicit with various conventions borrowed from the visual arts and unique disciplinary semiotic codes. Figure 2 illustrates three chemistry representations that represent the same structure in different ways. The ball-and-stick model uses pseudo-3D conventions to highlight spatial relationships explicitly, the dash-wedge perspective formula uses unique symbols to highlight only critical spatial relationships, and the line-angle diagram hides many spatial relationships and most of the atoms to emphasize the connectivity in a structural backbone.

In all of these representations, *expert chunks (*known as functional groups) are present. Akin to the bishop-and-knight-mate chunk, functional groups comprise a set of recurring patterns of atoms that encode important identity and spatial information. In the figure, the *hydroxyl* (−OH) functional group consists of one oxygen and one hydrogen in a specific bonding pattern (i.e., *sp*^3^ hybridization, bent geometry), whereas the *methyl* subunit (−CH_3_) consists of one carbon atom and three hydrogen atoms bonded a different pattern (i.e., *sp*^3^ hybridization, tetrahedral geometry). Experts learn to quickly identify these “chemical chunks” in a structure since these groups have unique chemical and physical properties important to their work. Many chemical structures also contain redundancies in the form of repeated elements inherent to the identity of a molecule, which may also be present in chunks as seen in the ball and stick representation of Fig. 2. The molecule contains 15 atoms represented by 15 spheres; however, only four unique elements are present, which are represented by the four associated colors. In the most abstract example, the bond line diagram does not explicitly show the redundant hydrogen atoms that are bonded to the carbon backbone, which experts readily perceive.

The various features of chemistry representations and the related disciplinary content provide a rich context for studying the role of domain-general and domain-specific strategies, spatial thinking, and visual working memory capacity in the development of expertise more broadly. Although prior work has revealed a diversity of strategies available for supporting spatial problem-solving in science disciplines, the cognitive processes and capacity limits underlying the encoding and transformation of the types of visual structure depicted in scientific representations has not been systematically examined.

Furthermore, whether individual differences in spatial skills are related to domain-general visual chunking strategies and whether that relationship can be accounted for by differences in visual working memory capacity are unclear. While visual working memory has been found to be related to measures of fluid intelligence (Fukuda, Vogel, Mayr, & Awh, 2010; Unsworth, Fukuda, Awh, & Vogel, 2014), to our knowledge, its relation to spatial skills has not been established. Because expert chunks in organic chemistry representations are characterized by both spatial properties (proximity) as well as visual properties (color conjunctions), the extent to which the development of expertise in the domain relates to the use of visual chunking strategies or spatial skills is unclear.

## Present study

Studies of individual differences in novice and expert performance have typically examined the relationship between performance and spatial skills (for novices) or the use of chunking strategies (for experts). In this study, we focus on novice participants, who had completed at least one semester of college-level chemistry (i.e., were enrolled at the time of this study in General Chemistry II or higher) and naive participants who had never been enrolled in a college-level chemistry course. Here, we attempt to identify whether these groups leverage spatial grouping or domain-general redundancy to encode disciplinary representations that include expert chunks that they have little to no experience using. If these participant groups are able to detect such regularities in disciplinary representations, such detection would provide initial evidence that disciplinary expertise can develop, in part, from the use of visual processes that are sensitive to the structure present in disciplinary representations. Moreover, in this study we aim to identify whether this sensitivity varies as a function of spatial skills or the dimensionality of the representation’s arrangement. Of the three types of visual structure chunking discussed above—domain-general redundancies, spatial groups, and expert chunks—we begin our exploration with the most general type, spatial groups, to test whether viewers are especially sensitive to the information present in those groups.

In both studies we employed a change detection paradigm (Luck & Vogel, 1997; Rouder, Morey, Morey, & Cowan, 2011). Participants were presented with a molecular representation (cue stimulus) that varied by number of informational units (i.e., colors) and whether changes occurred within or outside of spatial groupings. After a brief encoding period, participants were presented with a second molecular representation (target stimulus) and asked to determine if the cue and the target were identical or a mismatch. Mismatch pairs resulted from introducing a new color into the target that either maintained or changed the internal composition of spatially grouped units from the cue stimulus. Since these studies took place with chemistry novices and naive individuals, stimuli were based on ball-and-stick models of molecular structures in which each color corresponds to a unique element; these representations require less training in the discipline to interpret than other representations, such as dash-wedge diagrams that rely on more abstract diagrammatic conventions.

To date, the majority of stimuli used to probe visual working memory capacity have included only one- and two-dimensional arrangements. Given our interest in the development of expertise, we employed both two- and three-dimensional arrangements, which are more representative of the types of representations a novice or expert would encounter in the normal course of study in chemistry and other STEM disciplines. We therefore constructed stimuli as pseudo-3D images using ball-and-stick molecule representations whose arrangement extended into a 2D plane as well as structures whose arrangement extended into 3D.

**Fig. 3:**
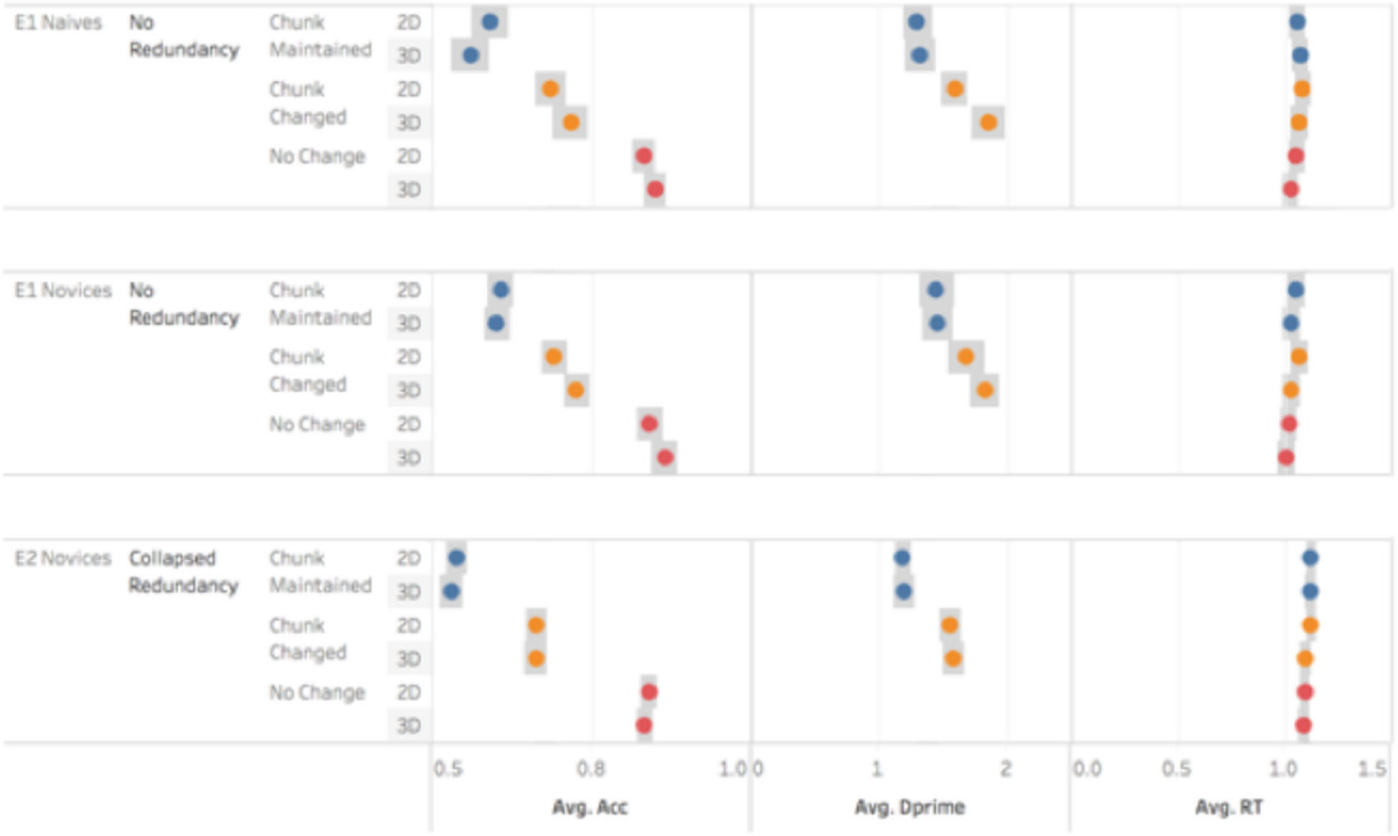
Accuracy, dprime, and response time across Experiments 1 and 2 (collapsed across redundancy manipulations). No reliable performance differences were observed between the 2D and 3D arrangements, but performance was reliably higher for chunk-changed trials compared to chunk-maintained trials for both accuracy and Dprime. Circle = group means, gray rectangles = standard error

To minimize the ability of participants to use verbal strategies (e.g., naming the colors or identities) to encode the stimuli, we included a dual task paradigm that applied an extraneous verbal load during encoding. Study 1 examined whether participant performance was sensitive to the presence of spatial groupings in these molecular representations. In Study 2, we counterbalanced the type of changes in target stimuli and the presence or absence of color redundancies (in addition to spatial groupings) in the cue stimuli. Our results across both studies (see Fig. 3) demonstrate that naive and novice students were sensitive to spatial groupings but, surprisingly, did not appear to leverage domain-general redundancies in the present displays. We also found that this sensitivity—and performance on the task more generally—was not associated with measures of spatial skills.

### Study 1

Study 1 aimed to identify whether and how naive and novice students perceive and encode expert chunks in disciplinary representations in the absence of extended disciplinary training. For this study, we employed common disciplinary representations from chemistry (ball-and-stick models). Participants were presented with a cue stimulus and asked to determine whether a target stimulus was identical to the cue or a mismatch. Each cue stimulus included eight units (i.e., colors that simulated individual atomic identities) for encoding and comparison but varied by geometry (2D, 3D). In mismatch targets, we varied the location of the color replacement so that it occurred either outside of, or within, a chunk. Given that the participants in this study are not experts, we predicted that participants would be better able to detect a mismatch when it occurred in a spatial group if they rely on a spatial grouping strategy. We made no prediction about the role of arrangement dimensionality on performance: Although we varied the spatial complexity of the items between 2D and 3D geometries, whether novices would treat them differently since all stimuli were rendered in pseudo-3D was not clear *a priori*.

The chunks we selected for these stimuli correspond to common functional groups ubiquitous in both organic and inorganic structures that chemists encounter routinely in the course of their work. Starting from a base set of eight colors, we constructed functional groups to include in the stimuli as described below. The functional groups represented 20% of the possible combinations of colors in the stimuli set. Arguably, individuals without chemistry expertise should encode all eight colors as individual units given their lack of knowledge about the chunks. However, if naive and novice participants are sensitive to the spatial grouping of the functional groups, they should display improved performance in trials where units in spatial groupings change as opposed to trials where the change occurs outside of a spatial grouping.

#### Methods

##### Participants

Forty-two undergraduate chemistry students (novices) and 25 undergraduate students with no chemistry background (naives) participated in Study 1. Each novice participant was recruited from the population of students enrolled in a first-semester organic chemistry at a Midwestern university. Naive participants were recruited from two other research universities— one in the Midwest and one in the West. Colorblind individuals were excluded from the study.

##### Materials

Study materials included digital molecular representations.

***Molecular representation stimuli*** Stimuli consisted of sequentially presented molecular models presented in pseudo-3D; stimuli were rendered in Jmol™, a digital drawing program that used shading and perspective to generate pseudo-3D images. Stimulus pairs were either identical (match) or differed by replacing one color in the cue stimulus (mismatch). All stimuli were constructed to have a single central element connected to other elements denoted as *ligands*. Ligands were chosen to create chemically meaningful representations consistent with established chemical principles and could be made of one, two, or three spheres (representing elements). The central element remained constant in each pair with color replacements occurring only in ligands.

Each stimulus contained four ligands connected to a central atom. All stimuli contained seven spheres across the four ligands. These spheres comprised two single-element ligands, one two-element ligand, and one three-element ligand. The two-element and three-element ligand in each stimulus were known expert chunks commonly found in textbooks and scientific journals. Two chunks were included in each stimulus to deter participants from focusing attention on a single multi-element ligand during the cue presentation. Two-dimensional stimuli were selected to be spatially located in a single horizontal plane; 3D stimuli were not constrained to coplanarity. Standard color conventions used in the Jmol™ modeling software used to make these stimuli were used to color code element identity. Twenty-four (24) unique molecules were created for each geometry (2D and 3D), giving 48 unique molecules.

**Fig. 4:**
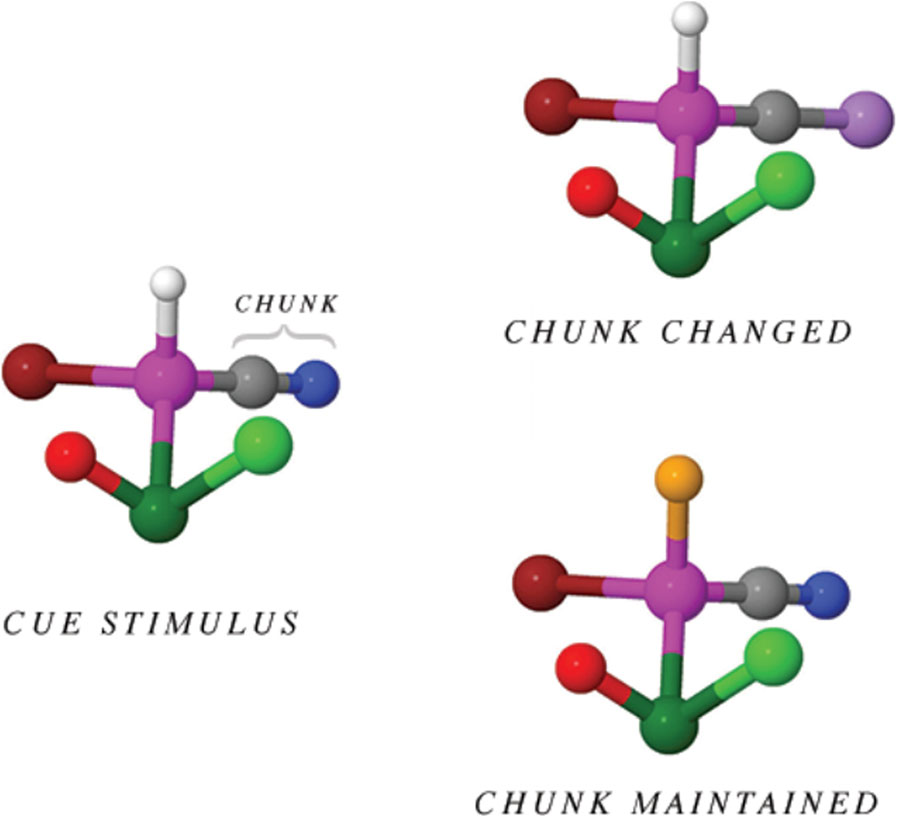
Examples of cue stimulus, chunk-changed stimulus, and chunk-maintained stimulus (all 2D) used in Study 1. Note that the relevant chunk in this example is the gray atom (carbon) and blue atom (nitrogen) indicated in the cue stimulus

Match targets contained stimuli that were identical to the cue stimuli. Mismatch targets were created in two ways: a new element not present in the cue stimulus replaced either (1) a randomly selected single-element ligand (chunk-maintained mismatch) or (2) a randomly selected atom from either the two- or three-element ligand (chunk-changed mismatch). Figure 4 illustrates the differences between a cue stimulus and the mismatch target types.

##### Procedure

Study 1 employed a within-subjects design. Participants made identity judgements about sequentially presented molecular stimuli. The order of the stimulus pairs was randomized for each participant. In each stimulus pair, the target stimulus was randomly rotated by 10 degrees to discourage comparison strategies based on local spatial correspondence. All rotations occurred in the picture plane.

Participants were first screened for colorblindness using the Ishihara test of color perception (Waggoner, 2005). Participants who incorrectly identified three or more items on this test were excluded from the study. The experiment was presented using PsychoPy v. 1.52.8. At the start of the experiment, participants viewed a series of instruction screens that described how to judge whether a stimulus pair was a match or mismatch. Participants were instructed to respond “match” or “mismatch” by keystroke as quickly as possible. Each trial began with a randomly generated verbal interference string of four consonants (e.g., “XFQL”) that participants were instructed to repeat aloud continuously throughout the trial. The cue stimulus in each pair was then displayed for 0.5 s before disappearing for 0.5 s to leave a white screen. The final stimulus in the pair was then shown for a maximum timeout duration of 2.5 s. Participants who did not respond within the timeout duration received an incorrect score for the trial and were instructed to respond faster on subsequent trials. Participants were randomly prompted (on 20% of all trials) to type in the verbal interference string to confirm adherence to the dual task instructions. Participants took 45–60 min to complete the procedure. Participants received either $20 USD or course credit for participation.

#### Results

For each participant group, accuracy and response time were analyzed via a 2 (dimension: 2D vs. 3D) × 3 (target: identical, chunk-changed, chunk-maintained) repeated-measures ANOVA. Sphericity could not be assumed for accuracy and response time; thus, a Greenhouse-Geisser correction was used when applicable. Although our intent was not to compare novice and naive students, a post hoc analysis revealed no significant differences between these groups in terms of accuracy (*F* (1,65) = .002, *p* = .9).

#### Novices

Overall accuracy on the verbal interference task was .90. We observed a main effect of target (*F* (1.25,51.38) = 52.2, *p* < .001, η_p_^2^ = 0.56). Participants were more accurate at identifying identical stimuli (*M* = 0.84, *SD* = 0.10) than chunk-changed (*M* = 0.70, *SD* = 0.15; *F* (1,41) = 24.44, *p* < .001) or chunk-maintained stimuli (*M* = 0.58, *SD* = 0.18, *F* (1,41) = 67.21, *p* < .001). Participants were also more accurate at identifying targets when the chunk was changed than they were when the chunk was maintained (*F* (1,41) = 90.47, *p* < .001). We observed a single interaction between dimensionality and target (*F* (1.86,76.37) = 4.20, *p* < .05, η_p_^2^ = 0.09). Accuracy for identical targets and chunk-maintained targets was greater for 3D stimuli than for 2D stimuli (*F* (1,41) = 53.78, *p* < .001).

For response time we observed a significant effect of the target condition (*F* (1.63,66.65) = 3.45, *p* = .047, η_p_^2^ = .078). Planned contrasts revealed only one significant difference: participants responded more rapidly to identical trials (*M* = 1.03, *SD* = 0.24) than to chunk-changed trials (*M* = 1.07, *SD* = 0.24).

Because we observed a positive response bias in the dataset, accuracy scores were converted to d' values (d' = z score _hit_ - z score _false alarm_). These values were analyzed as above via ANOVA. We observed a main effect of target (*F* (1,41) = 113.39, *p* < .05, η_p_^2^ = 0.73). Planned contrasts showed d' greater for chunk-changed stimuli (*M* = 1.72, *SD* = 0.11) than for chunk-maintained stimuli (*M* = 1.30, *SD* = 0.105, *F* (1,41) = 113.39, *p* < .05). We also observed an interaction between dimensionality and target type (*F* (1,41) = 8.286, *p* < .05, η_p_^2^ = 0.17). Participants were better able to identify chunk-changed targets in 3D stimuli (*M* = 1.85, *SD* = 0.129) than in 2D stimuli (*M* = 1.58, *SD* = 0.102), but no difference was observed between 2D and 3D stimuli for chunk-maintained targets.

#### Naive participants

For naïve participants, overall accuracy on the verbal interference task was .88. For accuracy, we observed no effect of dimensionality (*F* (1, 24) = 1.88, *p* = .18). In contrast, we observed a main effect of target (*F* (2, 48) = 48.73,*p* < .001, η_p_^2^ = 0.67). Participants were more accurate at identifying identical stimuli (*M* = 0.84, *SD* = 0.36) than chunk-changed (*M* = 0.60, *SD* = 0.49; *F* (1,24) = 35.75, *p* < .001) or chunk-maintained stimuli (*M* = 0.71, *SD* = 0.46, *F* (1,24) = 74.47, *p* < .001). Participants were also more accurate at identifying targets when the chunk was changed than they were when the chunk was maintained (*F* (1,24) = 21.54, *p* < .001). We observed no interactions in the data set. For response time we observed a significant effect of the target condition (*F* (2, 24) = 31.27, *p* < .001, η_p_^2^ = .57) and dimensionality (*F* (1, 24) = 43.77, *p* < .001, η_p_^2^ = .65). Planned contrasts revealed that participants responded faster for identical stimuli (*M* = 1.04, *SD* = 0.15) than for chunk-changed (*M* = 1.02, *SD* = .14; *F* (1,24) = 15.05, *p* = .001) or chunk-maintained stimuli (*M* = 0.71, *SD* = 0.46, *F* (1,24) = 47.99, *p* < .001). Participants responded more rapidly to 3D (*M* = 1.00, *SD* = 0.15) than to 2D stimuli (*M* = 1.02, *SD* = .17, *F* (1,24) = 47.99, *p* < .001).

We again observed a positive response bias in the dataset, which prompted an analysis of d' scores. We observed a main effect of target (*F* (1,24) = 19.72, *p* < .001, η_p_^2^ = 0.02). Planned contrasts showed d' was greater for chunk-changed stimuli (*M =* 1.74, *SD* = 0.63) than for chunk-maintained stimuli (*M* = 1.44, *SD* = 0.63). We did not observe an interaction between dimensionality and target type (*F* (1,24) = 1.76, *p* < .20).

#### Discussion

In Study 1 we observed better performance for identifying changes in a visual stimulus when the change occurred in a spatial grouping, or expert chunk, than when the change occurred elsewhere. Both accuracy and d' were higher when a mismatch resulted from the disruption of the color pattern in chunks present in the cue stimuli. Response time did not vary between the alternative mismatch conditions. Participants appeared to be better at detecting changes to chunks in the stimulus or at least find chunks more salient and preferentially encode them over non-chunk items. Notably, this was true for both novices (students in an organic chemistry class) and completely naive participants, indicating that domain knowledge is not necessary for detecting these chunks. This finding is consistent with other studies that have shown individuals have better memory for colors that co-occur repeatedly in a visual field (Brady et al., 2009), although color groupings were not systematically varied here. Interestingly, we did not observe a main effect of the arrangement dimensionality of the stimulus on performance. The interaction observed (greater accuracy for chunk changes in 3D molecules only) may indicate that the pseudo-3D nature of all the molecular representations biased participants to ignore the geometry present in the structures or that the chunks were detected on the basis of other features such as spatial proximity as opposed to the geometry of the stimulus.

Our finding that both novices and naive individuals are better able to detect changes in the chunks suggests that the individuals tested are relying on domain-general visual chunking strategies, i.e., spatial grouping, for encoding these disciplinary representations. These participants would not have been able to identify and encode the various spatial groups as expert chunks because their experience with the stimuli included here was limited (for novices) or nonexistent (for naive participants). Instead, these participant groups appear to perceive a chunk as a spatial grouping of information and encode it either preferentially or more efficiently. Given that these spatial groups do indeed correspond to expert chunks, both naive and novice students likely encode them selectively. This selective perception may inform the development of expert chunking strategies as disciplinary knowledge grows.

### Study 2

The results of Study 1 show that novices and naive individuals implicitly focus on common groupings of atoms that comprise expert chunks. In the case of Study 1, these expert chunks were also spatial groupings in that they allowed the compression of individual units based on their spatial proximity. In Study 2, we tested whether novices also leverage domain-general redundancies (color repetitions) to improve their performance with disciplinary representations. To do this we introduced the presence of a color redundancy in the stimuli on half of the trials. Thus, Study 2 examines how both domain-general redundancy and spatial grouping in disciplinary stimuli can influence visual working memory capacity in novices and how that contribution varies across two- and three-dimensional arrangement geometries. Performance was again measured by accuracy and response time. Based on the results of Study 1 that suggest the use of spatial grouping by naive and novice participants, we again predicted that novices would be better able to detect a mismatch when it occurred in a spatial grouping and that there would be no difference due to arrangement dimensionality. On the basis of prior evidence (Morey, Cong, Zheng, Price, & Morey, 2015), we predicted that accuracy would be improved for items with a redundant color present if they relied on domain-general redundancy. Given the lack of difference between naive and novice participants in Study 1, we did not include naives in Study 2.

Finally, in Study 2 we measured the spatial skills of the participants. Spatial skills have been found to be related to achievement in the organic chemistry domain (Harle & Towns, 2010), but we know little about the cognitive mechanisms that underlie this relationship. One possibility is that a proclivity to notice patterns in stimuli is a basic cognitive process that underlies spatial skill, and this is one mechanism by which highly spatial individuals have an advantage in encoding chemistry representations. If this is the case, we would expect highly spatial individuals to be faster to notice recurrent patterns, at least when they are characterized by spatial proximity (spatial groupings), and therefore to have better performance on our task, especially in detecting within-group changes. We tested this hypothesis in Study 2.

#### Method

##### Participants

Eighty undergraduate chemistry students participated in Study 2. Each participant was recruited from a population of students who had completed at least one semester of general chemistry at a Midwestern research-extensive university. Colorblind individuals were excluded from the study.

##### Materials

Study materials included two-factor referenced tests of spatial visualization ability: a cube comparison test and paper folding test (Ekstrom, French, Harman, & Derman, 1976), digital molecular representations rendered in Jmol™, and a survey of student attitudes and strategy choice.

***Cube comparison test*** The test consists of an untimed instruction page and two timed 21-question blocks for a total of 42 questions. Participants are allowed 3 min per block to answer as many questions as they can without sacrificing accuracy. All test items contain two six-faced cubes, resembling a set of children’s play blocks, where only the top, front, and right faces are visible. The visible faces display a letter or symbol, and participants are told that the hidden faces also contain any letter or symbol that is not a duplicate of those they see. Participants are instructed to mentally imagine reorienting one of the blocks and comparing it against the other block. Blocks that can be reoriented to align to one another are marked as “S” (same), and those that do not align are marked “D” (different). Each question has only one correct response.

***Paper folding test*** The test consists of an untimed instruction page and two 10-question blocks for a total of 20 questions. Timed administration of the paper folding test is identical to the cube comparison test. Test items depict a square piece of paper going through a series of one, two, or three folds that is then pierced by a pencil. The answer choices depict five unfolded pieces of paper with punched holes. Participants are instructed to select the piece of paper that represents the punched paper if it were unfolded in its current orientation. Each question has only one correct response.

**Fig. 5:**
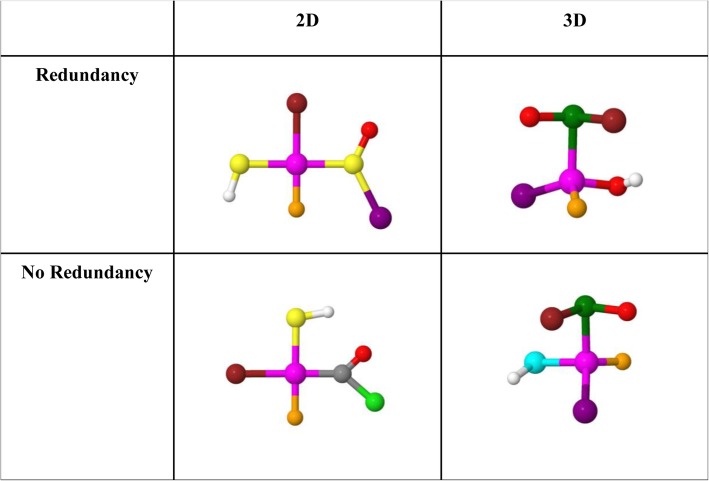
Examples of 2D and 3D stimuli with and without redundancy. Items with redundancy included two identical colors within the stimulus

***Molecular representation stimuli*** Stimuli were developed as in Study 1 with one modification, as shown in Fig. 5. In 50% of all cue stimuli, one redundant color is present in the structure. This resulted in half of the stimuli with seven unique colors among the ligands and half with six unique colors among the ligands. In mismatch trials we designed color replacements to ensure that (1) cue stimuli with a color redundancy maintained that redundancy in the target and (2) color redundancies were not introduced into targets if they were not already present in the cue stimulus. All colors present in any of the cue stimuli were replaced with equal probability, and redundancies were placed randomly in the structure. To limit the possibility that participants detected certain swaps because of the relative salience of initial or target color, or their relative difference in hue, swaps were counterbalanced to present equal occurrence of color swaps from source to target stimulus.

***Survey of student strategy choice*** We collected self-reports of strategy use among participants with a post hoc survey that asked participants to qualitatively describe how they made similarity judgments, to rate the difficulty of the tasks, and to provide demographic information. The survey was administered through the Qualtrics™ platform.

##### Procedure

The experimental portion for Study 2 was identical to that of Study 1.

Upon completion of the change detection task, participants completed the cube comparisons and paper folding tests. The order of administration for the psychometric tests was randomized. Participants then completed the self-report strategy survey. Only 50 of the participants were able to complete the survey given the allotted time for the entire experiment. Participants received either $20 USD or course credit (1% of total course points) for participation.

#### Results

Participant responses were scored as in Study 1. Overall accuracy on the verbal interference task was .88. Mean accuracy for the Cube Comparison and Paper Folding Tests were 24.29 (*SD* = 5.9) and 11.84 (*SD* = 3.7), respectively. First, accuracy was analyzed via a 2 (dimensionality: 2D, 3D) × 3 (change type: identical, chunk-changed, chunk-maintained) × 2 (redundancy: present, absent) repeated measures ANCOVA, controlling for the spatial skills measures. Neither the paper folding test (*F* (1, 77) = 1.09, *p* = 0.30) nor the cube comparison test (F (1,77) = .36, *p* = .55) were significant in the model. We observed only a main effect for the target type (*F* (1.52,115.68) = 12.191, *p* < .001, η_p_^2^ = 0.14). Planned comparisons of the target variable revealed that the average accuracy for identical targets (*M* = 0.84, *SD* = 0.01) was higher than for either chunk-changed targets (*M* = 0.66, *SD* = 0.02; *F* (1,77) = 11.87, *p* = 0.001) or chunk-maintained targets (*M* = 0.53, *SD* = 0.02; *F* (1,77) = 15.55, *p* < .001). Participants were also more accurate at identifying targets when the chunk was changed than they were when the chunk was maintained (*F* (1,77) = 125.98, *p* < .001). We observed no effects of the various factors and response time (Fig. 3, all F < 1).

d' was calculated as in Study 1 to correct for the observed positive response bias. A 2 (dimension: 2D vs. 3D) × 2 (target; chunk-changed vs. chunk-maintained) repeated-measures ANOVA revealed a main effect of target type (*F* (1,78) = 119.13, *p* < .05, η_p_^2^ = 0.604). As shown in the planned contrasts, on average, participants were significantly more sensitive to detecting changes in chunk-changed stimuli (M = 1.53, SD = .68) than chunk-maintained stimuli (*M* = 1.16, *SD* = 0.61, *F* (1,78) = 119.13, *p* < .05).

Participants’ self-report surveys were qualitatively coded for strategy use according to key words in each response. Table 2 lists the strategy types reported by participants. Participants reported a variety of strategies to make similarity judgements. Two strategies emerged as the most frequent. Eighteen percent of respondents reported searching for contrasting colors between stimuli in a mismatch. Interestingly, a similar fraction reported using a verbal encoding strategy to memorize a list of color names despite the dual task paradigm. Fourteen percent of respondents reported focusing their attention on a subcomponent of the structure and looking for changes only in that area. The same fraction reported that they focused their attention on the shape of the molecule and did not focus on the colors specifically. Twelve percent did not report a specific strategy or focused on the overall tone or pattern of colors and looked for changes in that tone. A few participants (8%) reported counting the number of colors in the stimuli to look for redundancies. Finally, two participants (4%) stated they interrogated a mental image without specifying how they identified matches or mismatches specifically.

**Table 2:** List of strategies reported by participants

Strategy	No. reporting	Example self-report
Contrasting colors	9	I thought it was easiest to identify differences if the color change was from a bright color, like yellow or green, to a darker color, like red or purple.
List of color names	9	At first, I tried to just memorize a list of three to four elements (colors), and if they changed, I would identify them as different molecules.
Piecemeal	7	Furthermore, I tried looking at certain sections of the molecule and memorized that color.
Focus on Shape	7	I also tried to memorize the formation of the structure by its orientation.
Unknown	6	I used the color change in the molecules.
Color tone/pattern	6	Saying the letters made it slightly confusing to really focus on the molecule and arrangement; therefore, instead of looking at bonds, I spent most of my time just looking at colors and the overall tone of the molecule
Redundancy	3	On the molecule, I looked for recurring colors because those stand out to me more than shapes and spatial arrangement.
Mental imagery	2	I would try closing my eyes between seeing two molecules so that the image would remind floating in my mind.
Count no. of colors	1	I tried counting the number of colors, not necessarily thinking about the colors themselves but while just thinking about how many occurrences there were of each color.

#### Discussion

In Study 2 we again observed improved performance for identifying changes in chunks relative to changes to other parts of the stimulus. Performance was improved both for accuracy and for discriminability with no differences in response time. This finding is consistent with our prediction that novices in chemistry can learn to encode chunks from simple repeated exposure to the spatial groupings. We also did not find a difference in performance between arrangement dimensionality, as predicted based on the results of Study 1. Contrary to our original prediction, we also did not find any benefit of color redundancy in the stimuli.

The lack of an observed benefit of color redundancy in Study 2 is surprising. Previous work has shown that even a single color redundancy in a stimulus provides a benefit for change detection (Morey et al., 2015). Here, perhaps the salience of the chunks overshadowed a strategy to rely on color redundancies. The location of redundancies present in 50% of the trials makes it likely that they were noticed, even if they were not relied upon for the task As shown in the self-report strategy survey, participants referenced noticing redundancies either by counting the number of colors or by looking for them explicitly.

The results of the self-report strategy survey demonstrate two important characteristics of the processes used for encoding disciplinary stimuli and identifying a change. First, and most importantly, no participants indicated that they explicitly used the spatial groups. This further validates our findings from Study 1 that the novices in our studies did not rely on expertise-based pattern recognition of the chunks to encode them. Second, the majority of participants did not explicitly acknowledge they were searching for relationships between the colors or for repeated colors in stimuli. This finding from the survey further supports our argument that detecting the changes in the expert chunks likely results from the implicit encoding of spatial groups and offers a partial explanation for why redundancies did not benefit the participants.

This study provided no evidence for the possibility that spatial skill is related to the ability to notice the spatial groupings that form chunks, in that spatial skills were not related to performance of the task. We also were surprised to observe no relationship between spatial skills and performance in this study for either of the spatial skills measures employed. However, although spatial skills are related to spatial working memory tasks (Shah & Miyake, 1996), this task engaged visual more than spatial working memory because the change to be detected was a visual rather than a spatial feature. We might expect a stronger correlation between experimental task performance and measures of spatial ability if the changes had consisted of element swaps, which would have increased the spatial nature of the task. While measures of visual working memory have been found to be correlated with measures of fluid intelligence (Fukuda et al., 2010; Unsworth et al., 2014). The relation between visual working memory and spatial skills has not been established to our knowledge and there is evidence for a dissociation between visual and spatial working memory (Darling, Della Sala, & Logie, 2007; Hecker & Mapperson, 1997).

## General discussion

In this paper we aimed to identify the processes by which early learners in a domain begin to acquire the exceptional working memory capacity displayed by experts. The majority of research on expert performance has focused on the role of domain knowledge and strategies resulting from extended practice in the domain (Chase & Simon, 1973). In contrast, most research on novices has focused on spatial skills as a gateway to expertise in STEM domains (Uttal et al., 2013; Wai et al., 2009). Here, we examined instead how chunking strategies might contribute to the development of expertise in a science domain. Prior research on expertise has demonstrated that people learn to identify domain-specific patterns among elements, and the visual working memory literature shows that people compress information by chunking redundant colors in a visual display. Our findings are consistent with previous research showing that individuals are sensitive to spatial groupings (i.e., expert chunks) in visual stimuli as ways of facilitating encoding and identifying changes in a stimulus. In two related studies we observed that novices (and naive individuals in one of the studies) perceived and encoded chunks present in chemistry representations. As seen in other studies (Brady & Alvarez, 2015a, b), the novices here learned to encode sets of colors in a representation that co-occurred in stimuli to more easily identify when one color was changed. Participants were more sensitive to changes in chunks than changes elsewhere in the stimuli after simple repeated exposure to such changes. Self-reports of participants in Study 2 indicate that they were not aware of the presence of these chunks despite improved performance on tasks that involved changes to the chunks. This suggests that they were used implicitly.

We outlined three possible visual chunking strategies that might contribute to performance: domain-general chunking, spatial grouping, and expert chunking (see Table 1). Although the results of both studies provide evidence that novices used spatial grouping strategies, we did not find that color redundancies improved performance. While prior work has shown that even a single redundancy can improve performance (Morey et al., 2015), we did not observe such an effect in Study 2. Participants were neither more accurate nor more sensitive to changes in stimuli when redundancies were present than when they were absent. These results are consistent with the spatial chunking hypothesis and not with the domain-general hypothesis. Moreover given that naive and novice participants showed similar performance, they indicate that semantic knowledge about the groupings (i.e. expert chunking) is not necessary to take advantage of spatial groupings).

Our analysis of participants’ self-reports suggests that only a few participants were aware of the redundancies, which indicates that the benefit of spatial groupings present in the stimuli may have suppressed any benefit of these color redundancies when they were present. An alternative study design comparing stimuli that contain only expert chunks to stimuli that contain only redundancies might identify the relative benefit of each feature; however, such stimuli would reduce the fidelity of the disciplinary representations and greatly diminish the ecological validity of the findings. At least, novices appear to rely on the spatial grouping in chunks in disciplinary representations, perhaps to bootstrap the encoding of information before they have learned domain semantics. This finding leads us to tentatively argue that both naive individuals and novices are able to leverage *spatial grouping* (see Table 1) to encode information presented in disciplinary representations as opposed to redundancies or chunks (only available to experts).

We also aimed to extend the research on perceptual strategies by varying the geometry of the stimuli. We observed no effect of arrangement dimensionality in either study, which suggests that these stimuli are encoded similarly. This finding is somewhat surprising given that the objects with 3D geometry are more spatially complex than the 2D objects. At the least, one might have expected the 3D objects to be more difficult to encode than the 2D objects. In contrast, the potential of any visual grouping strategy to compress information may have permitted participants to encode the 3D objects as efficiently as the 2D objects. Additional studies are needed that vary the disciplinary representations and more fully explore the relationship between visual working memory capacity and arrangement dimensionality.

A secondary goal of these studies was to identify whether these perceptual processes offer some insight into the differing achievement among STEM students of high and low spatial skills. Spatial skills often correlate with STEM achievement and persistence (Wai et al., 2009), but a causal account to explain this correlation is lacking. One possible hypothesis is that highly spatial students are better able to compress information in a disciplinary representation by leveraging chunks. We examined this hypothesis in Study 2 by including multiple spatial skills measures as a covariate in our models. We observed no relationship between spatial skills and any outcome measure in the study, which casts doubt on the validity of the hypothesis. Participants of varying spatial skills were able to identify changes in chunks equally well, and redundancies did not improve accuracy as discussed above. Alternatively, the change detection paradigm used here possibly does not recruit spatial skills because participants were not required to perform spatial transformations on the stimulus to complete the task and the change to be detected was visual (a color change), not spatial in nature. Whatever the role of spatial skills in STEM achievement, whether the encoding of chunks fulfills that role remains unclear from these studies.

Future investigations using these stimuli might investigate how spatial transformations affect performance as well as how naive and novice participants differ from real experts. The stimuli included in these studies were constructed using ligands that correspond to expert chunks in the domain. Whether naives, novices, or experts differentially employ domain-general visual chunking strategies for spatial groupings that are not domain-relevant would require participants to compare real chunks to “nonsense” chunks. Based on our results here, we predict that all three groups would selectively perceive and encode nonsense chunks, but we would expect experts to display improved performance for real chunks relevant to their domain and that experts may possibly underperform novices when changes occur outside of semantically meaningful chunks. Similarly, future studies might include stimuli that require participants to perform complex spatial transformations (e.g., rotations) on the objects while maintaining them in memory. Such a design would provide an additional method of investigating whether there is any relationship between spatial skills and the encoding of spatial groups as expertise develops.

Regardless of the role of spatial skills, our findings suggest that basic perceptual processes might be leveraged to improve success in the STEM curriculum. Typical curriculum models emphasize the semantic information encoded in disciplinary chunks; however, little time is devoted to helping students perceive these chunks among the various representations in a domain (Nathan, Stephens, Masarik, Alibali, & Koedinger, 2002). Educational interventions that aim to help learners identify patterns present in a STEM representation through simple repeated exposure have shown some success in mathematics (e.g., Perceptual Learning Modules as described by Kellman, Massey, & Son, 2010); medicine (Kellman, 2013); and, most recently, chemistry (Rau, 2018). Although such interventions do not appear to directly support conceptual change or skill acquisition in these domains, they do appear to support learners’ developing fluency with disciplinary representations. Arguably, such fluency is a component of expertise and contributes to the development of semantic knowledge and problem-solving. Early interventions that focus on recruiting domain-general processes may be an effective way to help learners, particularly low spatial skills learners, overcome existing barriers to entry in existing instructional models.

## Data Availability

Study data and materials are available upon request to the corresponding author.
